# Deciphering the Venomic Transcriptome of Killer-Wasp *Vespa velutina*

**DOI:** 10.1038/srep09454

**Published:** 2015-04-23

**Authors:** Zhirui Liu, Shuanggang Chen, You Zhou, Cuihong Xie, Bifeng Zhu, Huming Zhu, Shupeng Liu, Wei Wang, Hongzhuan Chen, Yonghua Ji

**Affiliations:** 1 Laboratory of Neuropharmacology and Neurotoxicology, Shanghai University, Nanchen Road 333, Shanghai 200444, P.R. China; 2 Department of Neurology, Tongji Hospital, Tongji Medical College, Huazhong University of Science and Technology, 1095 Jie Fang Avenue, Hankou, Wuhan 430030, Hubei, China; 3 Department of Pharmacology, Institute of Medical Science, Shanghai Jiao Tong University School of Medicine, South Chongqing Road 280, Shanghai 200025, P.R. China

## Abstract

Wasp stings have been arising to be a severe public health problem in China in recent years. However, molecular information about lethal or toxic factors in wasp venom is extremely lacking. In this study, we used two pyrosequencing platforms to analyze the transcriptome of *Vespa velutina*, the most common wasp species native in China. Besides the substantial amount of transcripts encoding for allergens usually regarded as the major lethal factor of wasp sting, a greater abundance of hemostasis-impairing toxins and neurotoxins in the venom of *V. velutina* were identified, implying that toxic reactions and allergic effects are envenoming strategy for the dangerous outcomes. The pattern of differentially expressed genes before and after venom extraction clearly indicates that the manifestation of *V. velutina* stings depends on subtle regulations in the metabolic pathway required for toxin recruitment. This comparative analysis offers timely clues for developing clinical treatments for wasp envenoming in China and around the world.

The fear of wasp stings has been spreading throughout several provinces of China in recent years, largely due to the powerful and deadly Asian black hornet, *Vespa* *velutina* (*V. velutina*), the most aggressive and fearful species in China (Fig. 1A and 1B). The envenomation of *V. velutina* can induce severe allergic or toxic reactions, resulting in organ failure and death.

Since 2006, with the extensive occurrence of thousands injured and dozens of deaths caused by wasp stings, it has become a severe public health concern in China. Most intensively in the summer of 2013, wasp attacks killed 42 people and injured 1,675 people in three cities in Shanxi Province^1^. The in-hospital mortality of victims was 5.1% calculated by a clinical report based on 1091 hospitalized wasp sting patients from 2009–2011 in the Hubei Province, an astonishingly high mortality when compared to death cased caused by other venomous animals in just one region^2^. Yet effective treatments for wasp stings are not available. Therefore, it is urgent that we understand the toxic components of wasp venom, in order to develop well-targeted therapeutics and reduce the lethality of wasp sting.

Wasp venom has long been thought to contain low molecular mass compounds, proteins and peptides, in which peptides constitute approximately 70% of the venom components^3^. Due to the difficulty of collecting natural venom from wasps, it is challenging to obtain sufficient amounts for traditional chromatographic separations. As a result, our knowledge of the composition of wasp venom is based on proteomic analysis from a limited number of species^4^,^5^,^6^. Even so, fewer than 60 proteins have been individually identified and characterized from the venom of these wasp species (www.uniprot.org/program/toxins). As a common wasp species in China, *V. velutina* has surprisingly not been extensively studied in its native environment and no reference could be found on its venom. The only documented clues came from the clinical characteristics of patients injured by wasp stings in Hubei province^2^. Most patients with multiple wasp stings suffer from multiple organ dysfunctions caused by toxic reactions rather than an anaphylactic reaction, which means that the wasp venom toxicity can be attributed to hemolytic, myotoxic, neurotoxic, vasodilatory, nephrotoxic and hepatotoxic enzymes^7^. The differential expression of certain components of wasp venom in the “active” state (before venom injection) versus the “replenishing” state (after venom injection) has never been investigated^8^.

To explore the genetic mechanisms underlying the toxinology of rampant wasp injuries in China, we took advantage of a high-throughput sequencing platform to uncover the global transcriptome of *V. velutina*. By pooling the gene repertoire expressed in the venom gland and comparatively analyzing gene expression before and after venom extraction, we have built a clear picture of toxin types, quantitative transcriptional abundance, single nucleotide polymorphism (SNP) patterns and metabolic pathways for toxin production. The transcriptome of *V. velutina*, as described here for the first time, will be greatly helpful in understanding the toxicology of wasp stings and provide guidance for medical treatments.

## Results and Discussion

### Global characterization of venom gland transcriptome of *V. velutina* through 454 sequencing

Since the genomes of wasps in the *Vespula* genus have never been resolved, we used a long-reads 454 GS-FLX+ sequencing system to characterize the global features of the transcriptome of *V. velutina* venom gland.

**Figure 1:**
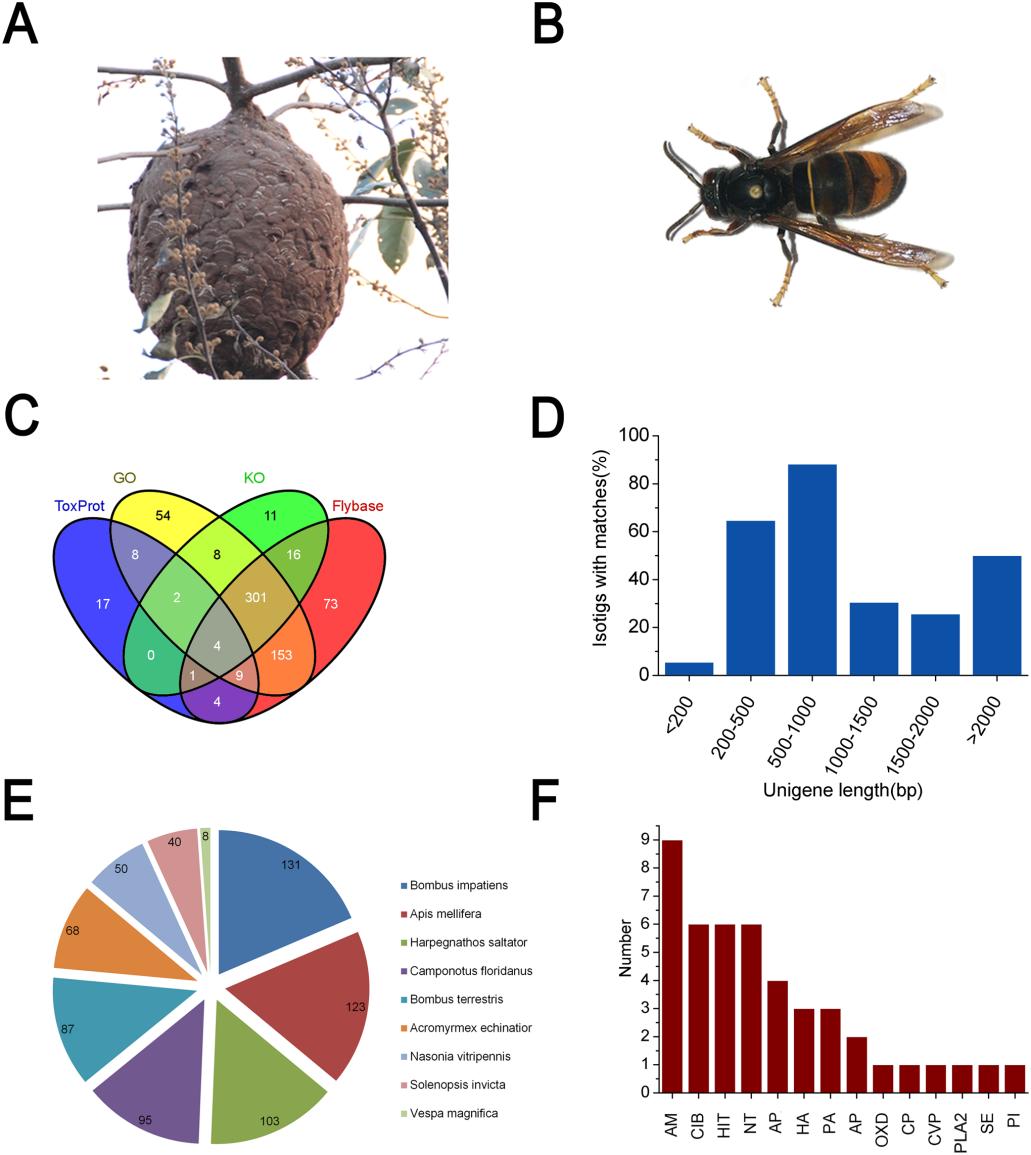
Morphology of the invasive wasp *V. velutina* and homology-based annotation of transcriptome. (A) Mature nest of *V. velutina* were found to be at the top of trees or under building eaves; (B) The morphology of *V. velutina* observed under the 40× microscope; (C) The intersection of the significant hits were based on number of isotigs annotated by blasting against GO, KO, Flybase and ToxProt (eval <1e-05, identity >30%, coverage >30%); (D) Taxonomic profile of the transcriptome. Number of top hits belonging to each species was indicated; (E) Percentages of size distribution of matched isotigs; (F) Number of top hits with significant homologous to the venomic proteins in ToxProt. Abbreviations: AM: Antimicrobial, CIB: calcium ion binding, HIT: Hemostasis impairing toxin, NT: Neurotoxin, APP: acid phosphatase, HA: Hyalurononglucosaminidase, PA: phosphatidylcholine 1-acylhydrolase, AP: Aminopeptidase, OXD: Oxidoreductase, CP: Carboxypeptidase, CVP: Cysteine-rich venom protein, PLA2: Phospholipase A2, SE: Serine esterase, PI: Protease inhibitor. The photographs of wasp *V. velutina* and their nest were taken by Chen SG.

**Figure 2:**
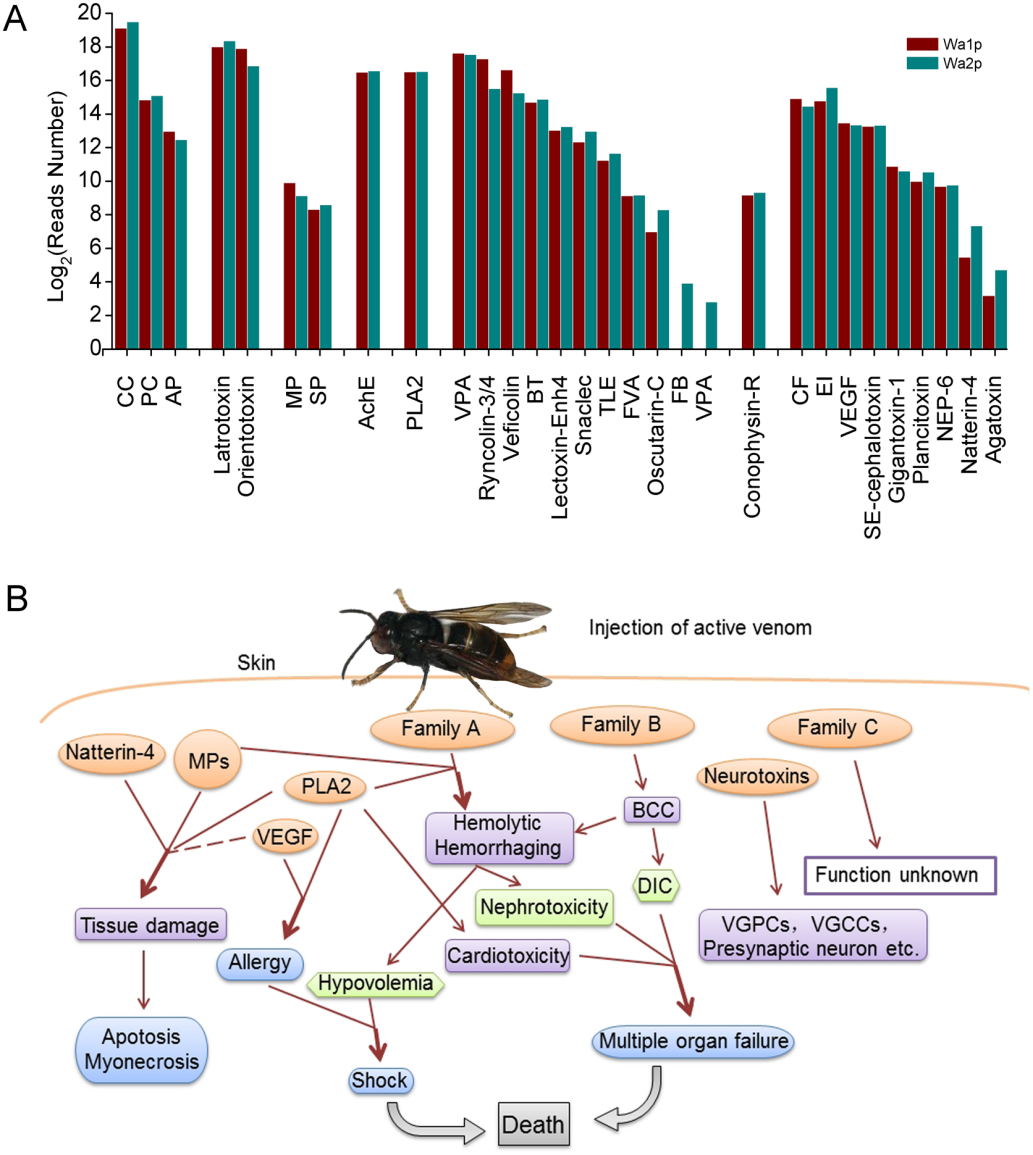
Comparison of number of reads participating in the putative toxins assembly and possible pathways of envenoming of *V. velutina* sting. Abbreviations for the associated putative toxins shown in (A): CC: Calcium channel; PC: Potassium channel; AP: Analgesic polypeptide; MP: Metalloprotease; SP: Serine protease; AchE: Acetylcholinesterase; PLA2: Phospholipase A2; VPA: Venom prothrombin activator; BT: Blarina toxin; TLE: Thrombin-like enzyme; FVA: Factor V activator; FB: Fibrinogenase brevinase; VPA: Venom plasminogen activator; CF: Coagulation factor; EI: Endopeptidase inhibitor; VEGF: Vascularendothelial growth factor toxin; NEP-6: Nematocyte expressed protein-6; Agatoxin: U8-agatoxin-Ao1a. The morphological photo of *V. velutina* was contributed by Chen SG.

*De novo* assembly produced 1210 isotigs, which can be grouped into 1075 isogroups (Table 1). Of note, the overall size of reads participating in the assembly was close to 199 Mb (309 Mb before clean), which is comparable to the only reported genome size of *Parasitoid* *nasonia* (239.8 Mb), a close wasp relative to *V. velutina*^9^. Using Nr TopHit annotation, we classified the top hits into 804 unigenes. The statistical diagram integrated the hits from all the databases (Fig. 2A), revealing that over 80% of the unigenes with open reading frames had significant hits in at least one of the databases (Fig. 1C). All the involved species belong to the Hymenoptera lineage, demonstrating the high quality of the assembly and *bona fide* annotation (Fig. 1E).

GOSlim analysis provided a preliminary view of gene categories for all the unigenes annotated. The dominant gene function categories were “Cellular process”, “Metabolic process”, “Cell part”, “Cell” (organelle), “Binding”, “Catalytic activity”, suggesting that protein synthesis and metabolic pathways may account for the major intracellular processes in *V. velutina* venom gland cells (Fig. S1A). KOG classification was used to predict the general functions of the unigenes from *V. velutina* venom (Fig. S1B). Generally, none of the clustered group had a portion with more than 10% and the largest group of unigenes was assigned to the cluster of “function unknown”, indicating that future studies are needed to characterize the components in *V. velutina* venom and to fully understand the toxicology of wasp stings. All the unigenes were searched against the KEGG pathway database to identify potential biological pathways that are most active in the venom of *V. velutina* (Fig. S1C), most of which were found to be engaged in toxin synthesis and performance.

Using a blastx search against ToxProt, we drew a preliminary picture of toxin types in *V. velutina* venom (Fig. 1F). A total of 45 sequences were toxin-like transcripts among all 1,075 isogroups, accounting for 4.18% of all the assembled transcripts, a higher proportion than is seen in some other common venomous species, e.g. scorpion (*Centruroides noxius*, 1.20%)^8^, snail (*Conus victoriae*, 1.45%; *Conus pulicarius*, 1.92%)^10^,^11^, but far less than in snakes (e.g. *Costa Rican*, 62.19%; *Crotalus adamanteus*, 11%)^12^,^13^.

### Detection of envenoming factors in *V. velutina* venom

Due to the limited number of annotated unigenes from 454 sequencing, efforts to understand the transcriptome of *V. velutina* venom gland were severely restricted. Meanwhile, a recent report indicates that some major components of scorpion venom are not identified at the transcriptional level^8^, possibly because venom at “resting state” does not contain the full spectrum of venom components. Therefore, we used a high-throughput illumina sequencing platform to further examine the transcriptome of *V. velutina* venom in the active state (Wa1p) versus the resting state (Wa2p).

**Figure 3:**
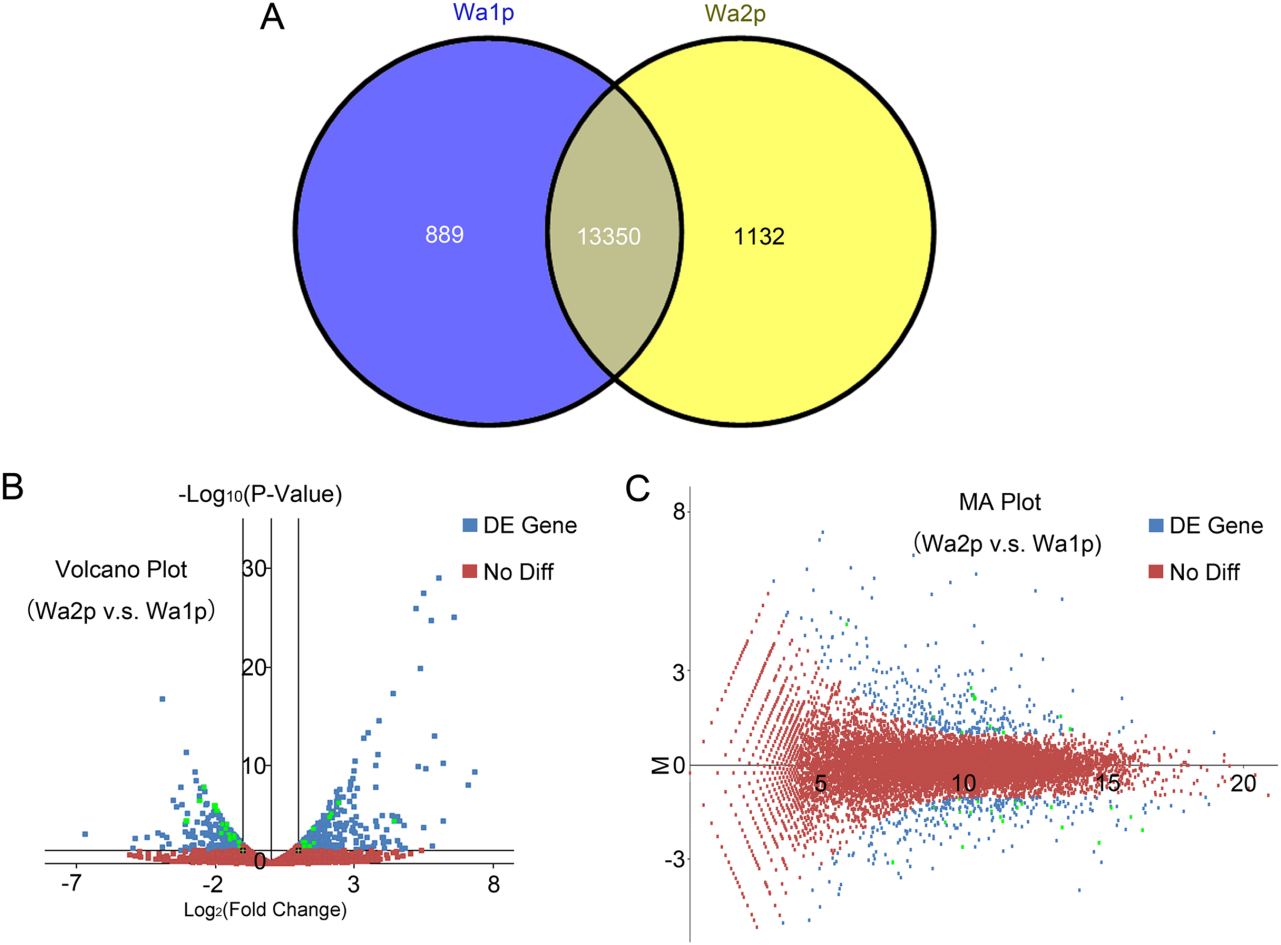
Differential expression of genes in resting and replenishing state of *V. velutina*. (A) Number of isogroups showing differential expression. The overlapping part of two circles indicates those isogroups equally expressed in both states. Wa1p: resting state. Wa2p: replenishing state. (B) Volcano plot indicates the gene expression levels in Wa1p and Wa2p groups. The y-axis represents the log_10_ significant of difference (P-value). The x-axis represents the log_2_ fold of changes. A fold change of log_2_ ratio ≥2 and p-value (significance of difference) <0.05 were set as threshold to determine the genes undergone differential expression. (C) MA plot indicates differential expression between Wa1p and Wa2p. The x-axis represents the abundance of expression, while the y-axis represents the difference between Wa1p and Wa2p. Genes with significant differential expression were shown in blue, among which genes selected for subsequent qRT-PCR validation were shown in green. Genes that were not differentially expressed between Wa1p and Wa2p groups were shown in red.

All the reads recorded were combined and assembled to draw a complete picture of the transcriptome of *V. velutina* venom. Among all the annotated unigenes, 11,685 (74.05%) and 6,595 (41.79%) unigenes mapped to the genome of *D. melanogaster* and *N. vitripennis*, respectively, reflecting a high level of species specificity^9^,^14^. When comparing unigene transcripts from Wa1p and Wa2p, 1,132 unigenes were abundant only in the active state, whereas 889 unigenes were specifically represented in the resting state (Fig. 3A). These results indicate that gene expression is up-regulated during the resting state in order to replenish venom lost during the active state.

The high-throughput sequencing data of *V. velutina* venom from Hiseq2000 in the present study enabled us to obtain the first complete view of venom composition in this wasp species in China, which can be used to address the question why human envenoming by *V. velutina* can result in severe toxic reactions and even death. Combined with the results from assembling the transcripts to Nr and ToxProt databases, 293 putative toxin-encoding sequences were identified and grouped into eight classes according to their possible working targets (Table 2). These included ion channel toxins, presynaptic neurotoxins, metalloprotease, serine esterase, phospholipase, hemostasis-impairing toxin, G-protein coupled receptor impairing toxins and other venom components whose function cannot be grouped into any class listed above. It remains to be established whether the toxic protein corresponding to each transcript can be efficiently translated with the putative toxicity intact.

Of all 293 putative toxins identified, hemostasis-impairing toxins accounted for the majority of venomic proteins in *V. velutina* (Fig. 2A, Table 2). This class of toxins comprised two families based on their mode of action. The first one includes toxic components that can induce a hemolytic effect or hemorrhaging, such as venom plasminogen activator, snaclec, lectoxin-Enh4, and fibrinogenase brevinase (Family A). Eight unigenes were classified into this family, three of which were matched well with snaclec (identity 32–35%), a snake C-type lectin from snake venom that binds specifically with activated factor X (FXa) and prolongs the blood-clotting time in vitro^15^. This kind of toxin can lead to severe nephrotoxicity and hypovolemia. The second one includes toxins that participate in the blood coagulation cascade (BCC), including factor V activator, oscutarin-C, ryncolin-3/4, veficolin, coagulation factor, thrombin-like enzyme and venom prothrombin activator (Family B). This family of toxins is also common in snake venom but rarely reported in other species, especially in Hymenoptera insects. One of the consequences of envenoming of these toxins can be disseminated intravascular coagulopathy, which often occurs in clinics^16^,^17^,^18^. This distinct coagulative effect, together with the hemolytic activity induced by the venom of *V. velutina*, were probably the major lethal factors of multiple organ failure^19^ (Fig. 2B).

Neurotoxins were the second largest group of putative toxins identified in the venom of *V.* *velutina*, and they can be divided into three groups: toxins that target ion channels including K^+^ and Ca^2+^ channels, toxins with presynaptic toxicity such as Latrotoxin and orientotoxin, and the serine esterase family including acetylcholinesterase. The main function of ion channel-specific toxins for *V.* *velutina* is to block ion channels of the prey or host defense. The targeted ion channels, especially Kv channels, are essential for the regulation of various physiological processes such as blood coagulation, fibrinolysis, and action potential transduction^20^. Notably, 85 unigenes showed homology to Latrotoxin, which was originally identified in the venom of black widow spiders (*genus Latrodectus*) and specifically targeted vertebrates through altering the presynaptic membrane permeability, causing Ca^2+^ overload within the nerve terminals and induced nerve degeneration^21^. This neurotoxic effect was similar to another putative presynaptic toxin in *V.* *velutina*, orientotoxin, from the venom of the giant hornet *Vespa orientalis*^22^. Finally, 22 unigenes in the venom of *V.* *velutina* were considered to be putative genes encoding acetylcholinesterase (AchE) which plays a critical role in cholinergic transmission by rapidly inactivating the neurotransmitter acetylcholine, leading to paralysis of the prey^23^ (Fig. 2B).

Protease is recognized as a common component in Hymenoptera venoms, which falls into the categories of metalloproteinase (MP) and serine protease (Table 3). Combined with Natterin-4, Phospholipase A2 (PLA2) and vascular endothelial growth factor toxin (VEGF-Tx), they were reported to induce or promote hemolysis and hemorrhaging. Apart from the direct toxic effects of a wasp sting injury, inflammatory reactions to some venom components can lead to organ failure as well^19^. Most importantly, MPs were also known to participate in the degradation of the extracellular matrix and contribute to both prey immobilization and digestion^24^. PLA2 was the only phospholipase with high transcriptional abundance in the venom of *V. velutina*, which was also commonly found in Hymenopteran venom^25^. Like other PLAs, PLA2 was able to disrupt the phospholipids of several types of biological membranes, leading to pore formation, cell lysis, inflammation and tissues damage^26^. VEGF-Tx, found in the venom of the social wasp *Polybia paulista* is known to promote vascular permeability, which may contribute to venom diffusion. The presence of VEGF-Tx can also stimulate the growth of cells from the venom glands and reservoir, preventing the rupture of the tissues due to the action of cytolytic components^27^. Finally, an ortholog of Natterin-4, a kininogenase identified in *Thalassophryne nattereri* fish venom, is also found in the venom of *V. velutina* at the transcriptional level, and is able to induce edema, nociception and cleave human kininogen and kininogen-derived synthetic peptides, releasing kallidin^28^ (Fig. 2B). Notably, PLA2 and VEGF-Tx in the venom of *V. velutina* are two allergens known to evoke immune reactions to human specific-IgE. However, as suggested by clinical studies, allergic reactions might not be the direct cause of death among patients who suffered from multiple wasp stings^2^.

### Venomic differential expression and functional classification of *V. velutina*

To determine whether differential expression of individual genes could be a hallmark of *V. velutina* venom before (Wa1p) versus after (Wa2p) venom extraction, we created two independent cDNA libraries to catalog functionally significant genes associated with toxic reactions and metabolism in the “replenishing” state of *V. velutina* venom (Table 3).

A total of 330 genes were significantly up-regulated after venom extraction while 300 genes were considerably down-regulated. In figure 3B, it showed that the maximum fold change of up-regulated genes for Wa2p v.s. Wa1p was higher than that of down-regulated genes in the venom of *V. velutina*. On the other hand, the Venn plot illustrating the relationship between gene expression abundance and difference between Wa1p and Wa2p indicated that the distribution of differential expressed genes was not correlated with the up/down regulated of genes in *V. velutina* venom (Fig. 3C).

GOSlim analysis for Wa1p and Wa2p indicated that there was no significant difference in the percentage of genes involved in each functional category (Fig. S2A), as was seen from the KOG classification (Fig. S2B). The consistency of gene expression profiles obtained through two sequencing methods suggested a high level of reliability for the two datasets, laying a basis for the subsequent pathway classification analysis of Wa1p and Wa2p.

The two sets of assembled unigenes from Wa1p and Wa2p were searched against the KEGG pathway database to compare the potential biological pathway distribution in the venom of *V. velutina* before and after venom extraction (Fig. S2C). There was little difference on the number of identified pathways between Wa1p and Wa2p. However, a prominent increase was observed in Wa2p in “Translation” and “Carbohydrate Metabolism” categories. By reciprocal mapping of the unigenes matched with these two KEGG categories for Wa1p and Wa2p, we found 92 unigenes in the “Translation” process and 45 unigenes in the “Carbohydrate Metabolism” process were specifically identified after venom extraction, more than that observed in Wa1p. This pattern of higher regulatory capacities for translation and carbohydrate metabolism was consistent with the previous observations in several other venomous arthropods: highly transcribed genes were involved in protein translation, glycolisis and lipid metabolism, possibly related to the regeneration of toxic peptides or enzymes in the venom during the “replenishing state”^29^.

To validate the quantitative results from the RNA-Seq experiments, we selected a subset of 26 putative toxins and 10 enzymes showing differential expression after venom extraction for analysis through qRT-PCR (Fig. S3). The representative genes were selected based on their expression levels according to the RNA-Seq data and also on their potentially regulatory roles in toxin synthesis and function.

Electrophoretic SDS-PAGE of the venom protein was carried out to confirm the transcriptome result (Fig. S6). The molecular weights of abundant venom proteins were shown to fall into the range of 37–70 kDa, which correlates well with the major envenoming factors detected in the *V. velutina* venom transcriptome, such as metalloproteinase (46 kDa), venom plasminogen activator (33 kDa), AchE (70 kDa), Ryncolin-3/4 (44 kDa) and coagulation factor (61 kDa). Other functionally significant toxins, like PLA2 (15 kDa) and VEGF-Tx (15 kDa) were less abundant at the protein level in the venom.

Combined, all the results were consistent with each other, supporting the validity of our transcriptomics data.

### Venomic SNPs of *V. velutina*

Despite the medical importance of developing treatments for wasp envenomation, few genetic markers have been identified for this life-threatening species. Without a whole-genome sequence for *V. velutina*, no intra-species SNP site could be retrieved. Hence, the genome of *N. vitripennis* and a combined transcript library of *V. velutina* venom from Hiseq2000 were used as two independent references to call SNPs existing in three *V. velutina* cDNA libraries.

**Figure 4:**
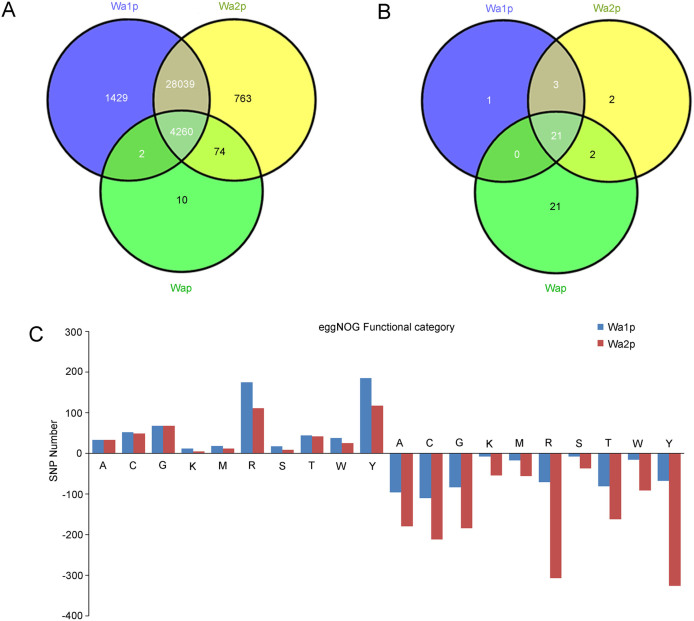
Venn Diagramed cSNP statistics in different groups (A) and number of SNPs in differential expressed genes belonging to different functional categories (B).(A): RNA processing and modification; (C): Energy production and conversion; (G): Carbohydrate transport and metabolism; (K): Transcription; (M): Cell wall/membrane/envelope biogenesis; (R): General function prediction only; (S): Function unknown; (T): Signal transduction mechanisms; (W): Extracellular structures; (Y): Nuclear structure; (Z): Cytoskeleton.

The number of SNPs identified by mapping to the *V. velutina* venom gland transcriptome (34,577) from Hiseq2000 was much higher than that identified by mapping to the *N. vitripennis* genome (50), suggesting a distant relationship between these two species even though they are classified into the same family. With the transcriptome of *V. velutina* venom gland from Hiseq2000 as reference, the Venn diagram showed a total of 4260 putative intra-species SNPs shared among all three samples (Fig. 4A). Inter-colony SNPs were also found between Wa1p and Wap (2), Wa2p and Wap (74). Wa1p and Wa2p shared 28039 SNPs, suggesting SNP sites were highly conserved among different colonies of *V. velutina*. Noticeably, the number of SNPs uniquely found in wasp samples at “resting” state was two- fold higher (Wa1p, 1429) than that at “replenishing” state (Wa2p, 763), whose underlying implications, such as whether it would contribute to diversified selection of toxins in *V. velutina* venom, remains to be validated in the future. Comparatively, SNPs identified with *N. vitripennis* genome were far less prominent due to the distant relationship between *V. velutina* and *N. vitripennis* (Fig. 4B).

To explore whether the unigenes that showed differential expression in Wa1p and Wa2p could be associated with the number of SNPs, we searched unigenes with SNPs against the eggNOG database. The number of SNPs in down-regulated genes in all functional terms were 2–5 fold higher than that in Wa1p, but fewer up-regulated genes were found. The most prevalent functional categories for SNPs were “Nuclear structure”, “General function prediction only” and “Energy production and conversion” (Fig. 4C).

## Conclusion

The Asian hornet is considered to be a dangerous invading predatory species, which has become a public health problem in China, and will likely become a public threat in its recently colonized countries. Until now, studies on *V. velutina* envenomation were less documented. With the help of high-throughput sequencing platform, a relatively intact gene repertoire in *V. velutina* venom gland was, for the first time, revealed. This study identified 293 putative toxin-encoding sequences, which were grouped into eight classes according to their possible working targets. Among all the toxins grouped, hemostasis-impairing toxins and neurotoxins represented the two largest families of venomic proteins in *V. velutina*. Meanwhile, through figuring out the functionally significant genes associated with toxic reaction and metabolism at the “replenishing” state of *V. velutina venom*, we identified genes with important regulatory capacities for protein translation involved in carbohydrate metabolism after venom extraction.

Collectively, this study provides first-hand genetic information for understanding the envenoming strategy of a dangerous wasp species. The knowledge on the venomics of this species could significantly benefit the decision-making for appropriate treatment strategies of stung patients.

## Methods

### Sample preparation

The wasp *V. velutina* was collected from the mountains at Jianshi County (30°06′–30°54′N, 109°32′–110°12′E), Hubei Province, China. The venom gland and reservoir of *V. velutina* were dissected from the rear apparatus of 10 individuals and homogenized in TRIZOL (Invitrogen, U.S.) in two different conditions: the replenishing state (no stimulus was performed before venom extraction) and the active state (three days after venom extraction with electric stimulation). Total RNA was extracted according to the manufacturer's protocol. RNA integrity was confirmed by the Agilent 2100 Bioanalyzer (Agilent Technologies) with clear characteristic peaks at 28S and 18S.

### cDNA library construction and RNA-sequencing

A cDNA library with long-reads 454 sequencing was constructed from the venom gland of *V. velutin*. The cDNA synthesis was performed with 3.5 μg of total RNA using cDNA Synthesis system kit (Roche) following the recommended protocol from the manufacturers. The first strand cDNA synthesis was primed with T7 oligo(dT) primers. After the second strand cDNA synthesis reaction, 5–10 ng of synthesized double stranded cDNA were amplified by in vitro transcription and the resulting products were purified using Agencourt AMPure XP Beads (Beckman). cDNA was nebulyzed to obtain fragments of 200–700 bp before sequencing. The cDNA fragments were subjected to ligation withthe sequencing adaptors provided in the GS FLX Titanium Rapid Library Preparation Kit (Roche), and small fragments were removed with AMPure XP (Beckman). The cDNA samples were end-repaired and adapter-ligated according to^30^. Streptavidin bead enrichment, DNA denaturalization and emulsion PCR were also performed as previously described^30^. Sequencing runs were performed using the GS-FLX with Titanium chemistry (Roche/454).

Two cDNA libraries of the *V. velutin* venom gland before and after venom extraction were constructed according to the Illumina manufacturer's instructions (TruSeq RNA Sample Prep Kit, Illumina). The mRNA was isolated using magnetic oligo(dT) beads. Fragmentation buffer was added to interrupt mRNA into short fragments (approximately 155 bp), and the short fragments were used as templates. Random hexamer-primers were used to synthesize first-strand cDNA. Buffer, dNTPs, RNase H, and DNA polymerase I were used to synthesize second-strand cDNA. Subsequently, short fragments were purified using a QiaQuick PCR extraction kit and resolved with elution buffer for end repair and poly(A) addition. The resulting short fragments were connected with sequencing adapters. The fragments with suitable range of lengths were selected based on the results of agarose gel electrophoresis were used as templates for library amplification. Library quantification was performed by Pico green and fluorescence spectrophotometer (Quantifluor-ST fluorometer, Promega; Quant-iT Pico Green dsDNA Assay Kit, Invitrogen). The library quality was confirmed by the Agilent 2100 Bioanalyzer (Agilent 2100 Bioanalyzer, Agilent; Agilent High Sensitivity DNA Kit, Agilent). Finally, the library was sequenced from both 5′ and 3′ ends using Illumina HiSeq 2000. Raw image data generated by sequencing was transformed by base calling into sequence data, which is called raw data/raw reads, and was stored in fastq format.

### Transcriptome assembly

Raw sequencing data from both 454 GS-FLX and Illumina HiSeq 2000 were filtered to remove low quality reads before subsequent analysis.

A global *de novo* assembly of the resultant reads from 454 GS-FLX+ was performed using Newbler 2.5 with the default parameters for EST analysis. Raw sequencing data from Illumina HiSeq 2000 was assembled with Trinity^31^. Unique identifiers were assigned to the reads according to the library and the sequencing system from which they were obtained.

### Annotation and qualitative analysis of the assembled unigenes

#### Annotation

To annotate all the unigenes obtained, we searched them against the protein databases NR, Swiss-Prot, KEGG, COG, Flybase (A Database of *Drosophila* Genes & Genomes, http://flybase.org/) and animal toxin database ToxProt (http://www.uniprot.org/program/Toxins) using blastx (BLAST, the basic local alignment search tool). The cut-off criteria were: E-value<10^−5^, identity percentage >30% and coverage >30%. The best matched hits were used to determine the sequence direction of each unigene. The priority annotation order was NR, Swiss-Prot, KEGG, COG, Flybase and ToxProt. If a unigene could not be aligned to any of the above databases, the software Getorf was introduced to determine its sequence direction. The blastp outputs were also used to obtain the gene ontologies with Blast2Go^32^.

#### Quantitative analysis of differentially expressed unigenes

To detect differentially expressed unigenes in *V. velutin* before and after venom extraction, the number of reads assigned to a gene was accounted and statistically compared. The package DESeq was adopted to estimate variance-mean dependence in the count data from Illumina sequencing assays and test for differential expression based on a model using the negative binomial distribution: *v* = *sμ* + *αs*^2^*μ*^2^, where μ is the expected normalized count value (estimated by the average normalized count value), *s* is the size factor for the sample under consideration, and α is the dispersion value for the unigene under consideration^33^.

#### SNP sites detection

Samtools were applied to export reads aligned to transcriptome or genome reference in mpileup format^34^. VarScan with a robust heuristic/statistic algorithm was used to call SNPs within each wasp sample that meets desired thresholds for read depth, base quality, variant allele frequency, and statistical significance^35^. Filtering threshold was set as following: read depth no less than 10, quality score no less than 20. The default parameter was used for quality control of flanking sequences in the step of “mpileup”.

### Quantitative real-time PCR validation

Total RNA of *V. velutina* at two stages (before and after venom extraction) was extracted as described above. Reverse transcription of each RNA sample was performed to get cDNA using the PrimeScript RT reagent Kit (TaKaRa) with an OligodT primer in a 10 μl reaction system. A total of 26 toxin-like and 10 transcripts related to toxin synthesis or performances were selected for qRT-PCR analysis. The qRT-PCR was performed in triplicates using the SYBR Premix ExTaq Kit (TaKaRa), in which β-actin was used as an internal standard. Primers used for this study were shown in Table S1. Absolute concentrations of target genes of two samples were determined using the standard curve quantitation method. Statistical analyses were performed using the least significant difference (LSD) test at P = 0.01 and P = 0.05 with DPS statistical software.

## Supplementary Material

Supplementary Information Supplementary Information

## Figures and Tables

**Table 1:** 454 Sequencing assembly statistics

Raw sequences	
Trimed Reads (Length > = 20 bp)	458,093
Raw Bases	309,753,616
Clean Bases (bp)	198,621,055

Reads Assembly Status	
Assembled	320,307
Partially Assembled	68,684
Singleton	52,516
Repeat	3,287

Assembly	
Isogroup	1,075
Isotig	1,210
Contig	1,759

Isotigs	
Sequence No.	1,229
Min Length	53
Max Length(bp)	11,260
Avg Length(bp)	894.87
N50 Length(bp)	1,020
Length > N50	338

**Table 2:** Illumina Assembly statistics

Raw Sequences	
Wa1p paired	47,035,303
Useful Reads %(Q20)	92.41%
Wa2p paired	54,962,972
Useful Reads %(Q20)	90.77%

Assembly Statistics(Contig)	
Total Length(bp)	53,583,487
Sequence No.	133,260
Max Length(bp)	28,415
Ave Length(bp)	402.097306
N50	693
GC %	34.51%

Assembly Statistics(Transcript)	
Total Length(bp)	195,858,761
Sequence No.	107,915
Max Length(bp)	38,922
Ave Length(bp)	1,815
N50	3,810
GC %	34.93%

Unigene Statistics	
Total Length(bp)	32,984,767
Sequence No.	15,780
Max Length(bp)	38,922
Ave Length(bp)	2,090
N50	3,068
GC %	35.75%

**Table 3:** Toxin families identified in *Vespa velutina*

Toxin Family		Unigenes	Reads	%Identity
**Ion channel toxins**	Potassium channel	14	29,217/35,073	39–58%
	Calcium channel	5	565,473/731,478	36–41%
	Analgesic polypeptide	1	7,988/5,636	50%
**Presynaptic neurotoxin**	Latrotoxin	85	226,484/293,056	30–61%
	Orientotoxin	1	243,360/119,828	53%
**Protease**	Metalloprotease	21	11,677/10,850	30–55%
	Serine protease	6	316/383	32–61%
**Serine esterase**	Acetylcholinesterase	22	40,857/36,312	30–52%
**Phospholipase**	Phospholipase A2	5	93,304/93,915	50–56%
**Hemostasis impairing toxin**	Venom prothrombin activator	52	157,343/130,723	30–60%
	Blarina toxin	8	21,260/23,767	30–59%
	Ryncolin-3/4	10	159,008/46,879	34–56%
	Thrombin-like enzyme	5	1,548/1,778	30–62%
	Snaclec	3	1,787/3,341	32–35%
	Factor V activator	3	560/573	31–34%
	Lectoxin-Enh4	3	318/274	34–41%
	Fibrinogenase brevinase	1	0/15	39%
	Oscutarin-C	1	126/312	46%
	Venom plasminogen activator	1	0/7	44%
	Veficolin	1	100,367/38,809	51%
	Vascular endothelial growth factor toxin	3	11,251/10,364	33–35%
	Coagulation factor	9	30,106/21,182	30–34%
**G-protein coupled receptor impairing toxin**	Conophysin-R	4	574/641	42–47%
**Other venom components**	Endopeptidase inhibitor	10	9,088/12,957	31–54%
	Plancitoxin	2	1,007/1,493	38–49%
	Gigantoxin-1	3	1,884/1,547	50%
	SE-cephalotoxin	9	8,187/8,843	30–41%
	U8-agatoxin-Ao1a	1	9/26	45%
	Nematocyte expressed protein-6	3	826/861	40–44%
	Natterin-4	1	44/160	38%
**TOTAL**		293	1,723,969/1,631,083	
