# Supplementary material for: Deciphering the Venomic Transcriptome of Killer-Wasp Vespa velutina
Source: Sci Rep. 2015 Apr 23;5:9454. doi: 10.1038/srep09454 (PMC5381768; doi:10.1038/srep09454)
Supplement: Supplementary Information [file srep09454-s1.pdf]

Supplementary Information

Deciphering the Venomic Transcriptome of Killer-Wasp *Vespa velutina*

Zhirui Liu, Shuanggang Chen, You Zhou, Cuihong Xie, Bifeng Zhu, Huming Zhu, Shupeng Liu, Wei Wang, Hongzhuan Chen, Yonghua Ji

Supplementary Figure legends:

Fig. S1: Annotation of 454 FLX+ Unigenes in GO, COG and KEGG database.

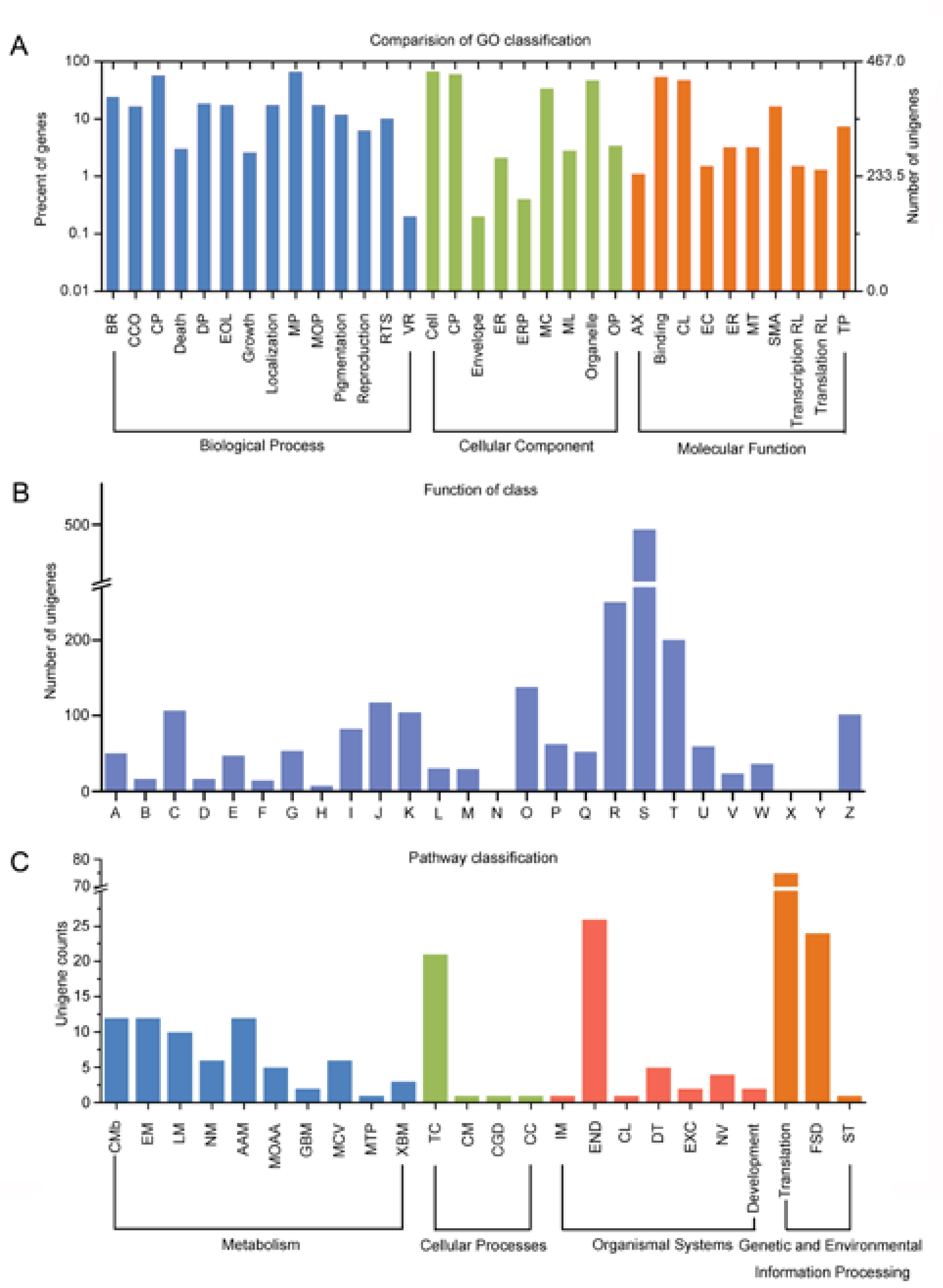

A) GO categories of the unigenes: BR: biological regulation; CCO: cellular

component organization; CP: cellular process; Death: death; DP: developmental process; EOL: establishment of localization; Growth:growth; Localization: localization; MP: metabolic process; MOP: multicellular organismal process; Pigmentation: pigmentation; Reproduction: reproduction; RTS: response to stimulus; VR: viral reproduction; Cell: cell; CP: cell part; Envelope: envelope; ER: extracellular region; ERP: extracellular region part; MC: macromolecular complex; ML: membrane-enclosed lumen; Organelle: organelle; OP:organelle part; AX: antioxidant activity; Binding: binding; CL: catalytic activity; EC: electron carrier activity; ER: enzyme regulator activity; MT: molecular transducer activity; SMA: structural molecule activity; Transcription RL: transcription regulator activity; Translation RL: translation regulator activity; TP: transporter activity;

B) COG classification of the unigenes.

A: RNA processing and modification; B: Chromatin structure and dynamics; C: Energy production and conversion; D: Cell cycle control, cell division, chromosome partitioning; E: Amino acid transport and metabolism; F: Nucleotide transport and metabolism; G: Carbohydrate transport and metabolism; H: Coenzyme transport and metabolism; I: Lipid transport and metabolism; J: Translation, ribosomal structure and biogenesis; K: Transcription; L: Replication, recombination and repair; M: Cell wall/membrane/envelope biogenesis; N: Cell motility; O: Posttranslational modification, protein turnover, chaperones; P: Inorganic ion transport and metabolism;

Q: Secondary metabolites biosynthesis, transport and catabolism; R: General function prediction only; S: Function unknown; T: Signal transduction mechanisms; U: Intracellular trafficking, secretion, and vesicular transport; V: Defense mechanisms; W: Extracellular structures; Y: Nuclear structure; Z: Cytoskeleton

C) KEGG pathways of the unigenes

CMb: Carbohydrate Metabolism; EM: Energy Metabolism; LM: Lipid Metabolism; NM: Nucleotide Metabolism; AAM: Amino Acid Metabolism; MOAA: Metabolism of Other Amino Acids; GBM: Glycan Biosynthesis and Metabolism; MCV: Metabolism of Cofactors and Vitamins; MTP: Metabolism of Terpenoids and Polyketides; XBM: Xenobiotics Biodegradation and Metabolism; TC: Transport and Catabolism; CM: Cell Motility; CGD: Cell Growth and Death; CC: Cell Communication; IM: Immune System; END: Endocrine System; CL: Circulatory System; DT: Digestive System; EXC: Excretory System; NS: Nervous System; Development: Development; Translation: Translation; FSD: Folding, Sorting and Degradation; ST: Signal Transduction.

Fig. S2: Anotation of Illumina Unigenes annotation in GO COG and KEGG database.

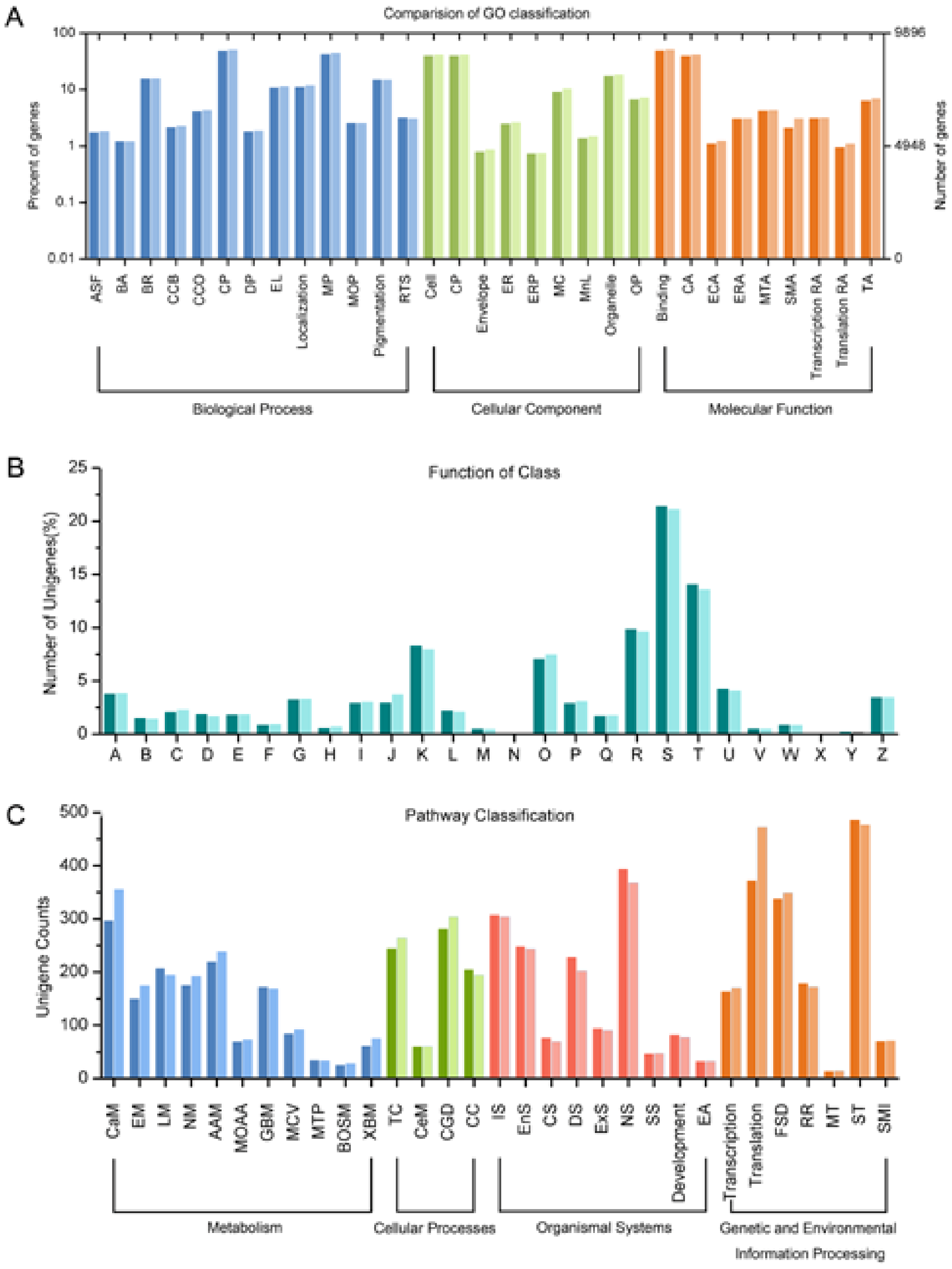

The percentage of unigenes in Wa1p and Wa2p were presented in deep and light colors, respectively.

A. GO categories of the unigenes.

ASF: anatomical structure formation; BA: biological adhesion; BR: biological regulation; CCB: cellular component biogenesis; CCO: cellular component organization; CP: cellular process; DP: developmental process; EL: establishment of localization; Localization: localization; MP: metabolic process; MOP: multicellular organismal process; Pigmentation: pigmentation; RTS: response to stimulus Cell: cell; CP: cell part; Envelope: envelope; ER: extracellular region ERP: extracellular region part; MC: macromolecular complex; ML: membrane-enclosed lumen; Organelle: organelle; OP: organelle part; Binding: binding; CL: catalytic activity; EC: electron carrier activity; ER: enzyme regulator activity; MT: molecular transducer activity; SMA: structural molecule activity; Transcription RL: transcription regulator activity; Translation RL: translation regulator activity; TP: transporter activity.

B.COG classification of the unigenes.

A: RNA processing and modification; B: Chromatin structure and dynamics; C: Energy production and conversion; D: Cell cycle control, cell division, chromosome partitioning; E: Amino acid transport and metabolism; F: Nucleotide transport and metabolism; G: Carbohydrate transport and metabolism; H: Coenzyme transport and

metabolism; I: Lipid transport and metabolism; J: Translation, ribosomal structure and biogenesis; K: Transcription; L: Replication, recombination and repair; M: Cell wall/membrane/envelope biogenesis; N: Cell motility; O: Posttranslational modification, protein turnover, chaperones; P: Inorganic ion transport and metabolism; Q: Secondary metabolites biosynthesis, transport and catabolism; R: General function prediction only; S: Function unknown; T: Signal transduction mechanisms; U: Intracellular trafficking, secretion, and vesicular transport; V: Defense mechanisms; W: Extracellular structures; Y: Nuclear structure; Z: Cytoskeleton.

C.KEGG pathways of the unigenes

CMb: Carbohydrate Metabolism; EM: Energy Metabolism; LM: Lipid Metabolism NM: Nucleotide Metabolism; AAM:Amino Acid Metabolism; MOAA: Metabolism of Other Amino Acids; GBM: Glycan Biosynthesis and Metabolism; MCV: Metabolism of Cofactors and Vitamins; MTP: Metabolism of Terpenoids and Polyketides; BSM: Biosynthesis of Other Secondary Metabolites; XBM: Xenobiotics Biodegradation and Metabolism; TC: Transport and Catabolism; CM: Cell Motility; CGD: Cell Growth and Death; CC: Cell Communication; IM: Immune System; END: Endocrine System; CL: Circulatory System; DT: Digestive System; EXC: Excretory System; NV: Nervous System; SS: Sensory System; Development: Development; EA: Environmental Adaptation; Transcription: Transcription; Translation: Translation FSD: Folding, Sorting and Degradation; RR: Replication and Repair; MT: Membrane

Transport; ST: Signal Transduction; SMI: Signaling Molecules and Interaction.

Fig. S3: Relative quantification analysis of 36 differentially expressed unigenes in Wa1p and Wa2p.

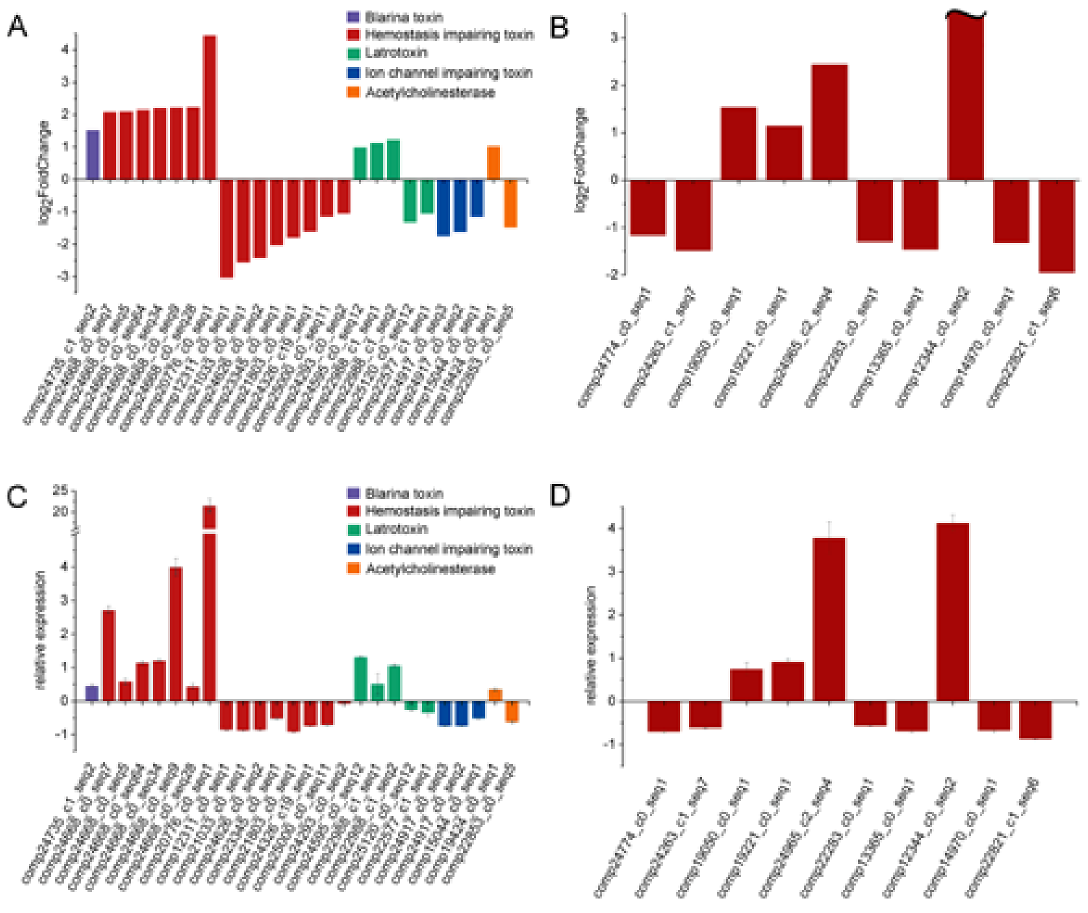

Log_2Fold of changes for the 26 putative toxins (A) and 10 enzymes (B) considered to be differential expressed after venom extraction; (C-D) qRT-PCR validation for expression level of the above unigenes.

Fig. S4: Reads quality and length distributions of transcriptome of *V. velutina* venom sequenced by 454 GS-FLX+.

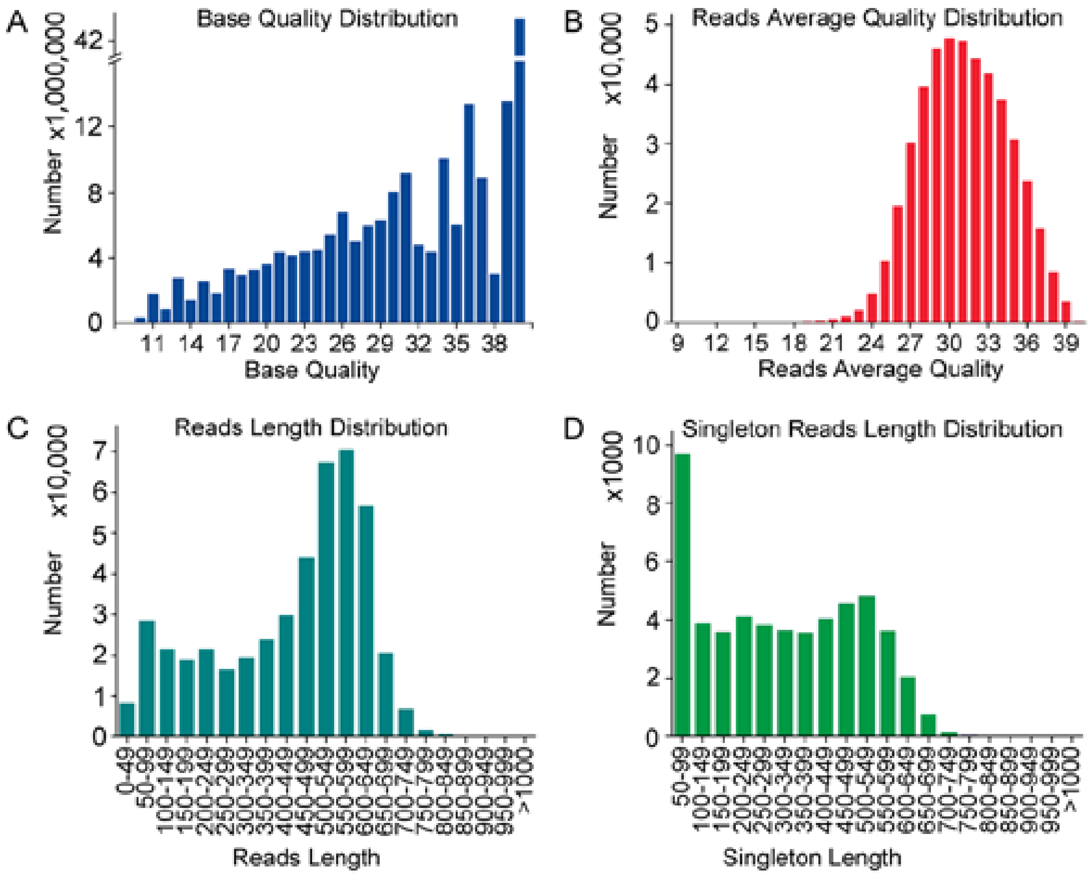

Fig. S5: Quality control and sequence content for reads obtained from Hiseq2000.

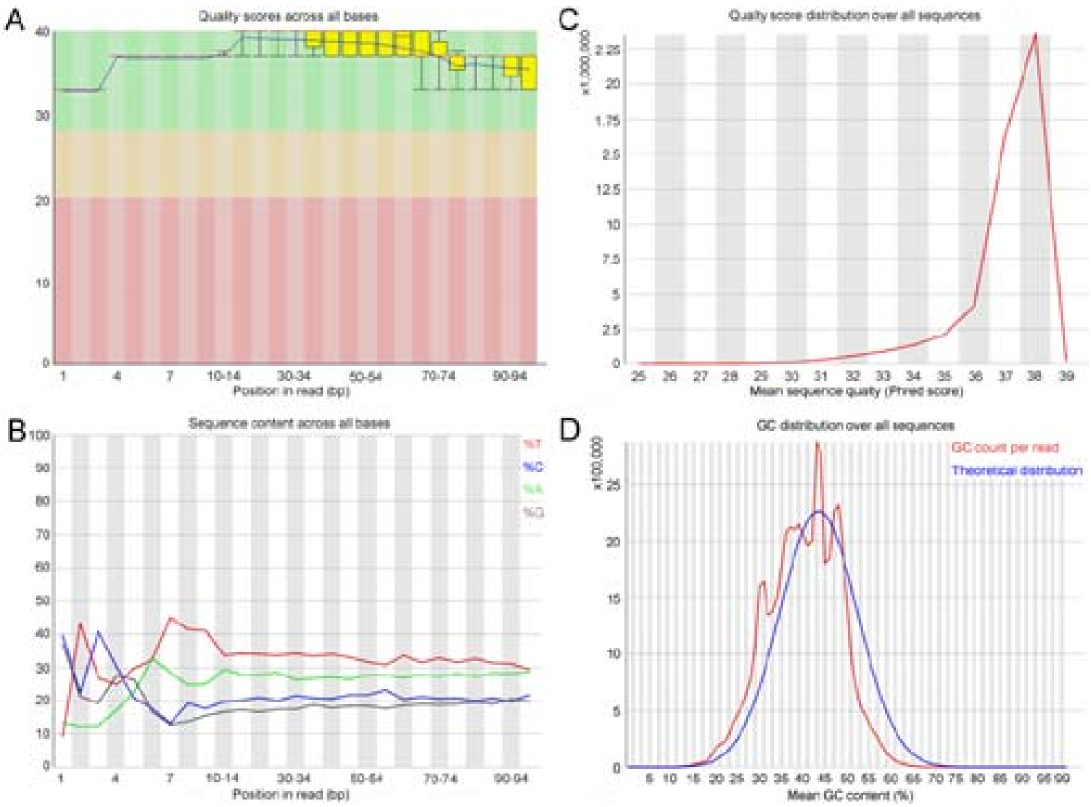

Fig. S6 Electrophoretic analyses of venom proteins.

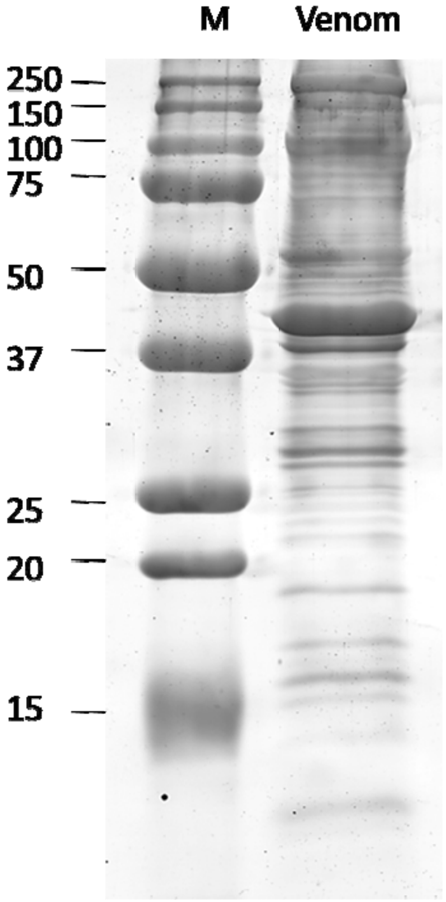

Venom reservoirs of *V. velutina* were dissected in PBS buffer supplemented with a protease inhibitors cocktail (PI; Roche) and residual tissues were removed by centrifugation. The total protein content of 20 µg venom reservoirs was run on a 10% SDS–PAGE under reducing conditions (right). The separated proteins were visualized by Colloidal Coomassie staining. Molecular weight standards (left) were in kDa.

Supplementary table:

Table S1: Primers used for qRT-PCR validation of putative toxin unigenes （No.1-26）and enzymes for toxins synthesis or performances （No.27-36）

No.	Primer	Sequence(5’-3’)	Location(5’)	Product(bp)
1	S	CTCATTTTGTGCATACATGACATAC	377	218
	A	TTCTTTTGGTTCTGGATGTGC	595	
2	S	GGAGGGATTATAGTCGAAGCTAG	223	339
	A	AGCAACCTTGGCCCATAGAA	562	
3	S	TTCAAACGACGATACCTATGAC	2097	133
	A	CCTACACGGCATACCGCTATT	2230	
4	S	CACAAGGTAGTTTTTCCTGTCTT	1406	161
	A	CTCCAGCAATATTTCTACACTCTTC	1567	
5	S	AGAGGATTGCCGAGACATAGAC	2247	196
	A	TTTGTATCCTATTCGCATGAG	2443	
6	S	GCAGAGGGGAGGGATTATAGTC	216	146
	A	AGACCTTGTTCGACTCTAGGA	362	
7	S	CACAAGGTAGTTTTTCCTGTCTTA	1656	183
	A	CACTTAATATCACACTCATAAGCTC	1839	
8	S	AACATGATGAAACTCATTCTCTTCG	1	174
	A	ATTACTTGAAGTTAAACCCCATTGC	174	
9	S	AACGAACAAGAAACGCATAATG	84	162
	A	AACAATGAAGCGCAGTAATGAC	246	
10	S	CCTTCCTCTCGATCAAACAAAC	203	224
	A	ATTCCTATTTGAGTGCGATTGA	427	

11	S	CTTGGACGACCGTGTATAACT	469	191
	A	GACGAAGCATAGAATGAGAATC	660	
12	S	AACGACGAGGACGACAATCTT	474	227
	A	TTGGAACGGATCTCAGTCTCAC	701	
13	S	AGGCAAAGGAAACATCGACTTAG	176	147
	A	TTGTTGGGGGGATAGATGACGA	323	
14	S	CCATAATATGAAGTAAAGAGAATCC	1150	170
	A	GGCAAGGTCTTACTGGAAATG	1320	
15	S	TACCTTCGCAAAGATCCACTC	115	182
	A	TGATGAAAACTTCAACTTGGG	297	
16	S	ACACATACGATTACATTGGGACC	2626	149
	A	CGACCGATTAGTACAGATTTGATT	2775	
17	S	CAAGAGATACGAGGTGGCGTTT	963	169
	A	CACGTCTGTGATCATCGTTCGA	1132	
18	S	ACCTTGTTCTATCAAATACTCTGC	2423	212
	A	CTTTCTATAGTAAAGTGTCTTGTGG	2635	
19	S	TGCTATAATTACATGTTTGACAAAG	1539	191
	A	CTTTCTATAGTAAAGTGTCTTGTGG	1730	
20	S	AAGTTTTAGTGCTGTTCAATGC	1184	236
	A	ACATTTGTACCACCTTATAGATACG	1420	
21	S	GTTCACTTTAACGACCTGACC	1332	179
	A	GATCTCCACAGAGATTTCCAC	1511	
22	S	CTTTTTCTTCTCAAGTTGTCAAGG	233	178
	A	GCTGGCATATTTATTCATCCC	411	
24	S	ACCAGTTGCCTCAAACGATTA	677	188
	A	GATCCATTCGAGATTAAGGAAAA	865	

25	S	GATCGAGACGAAGTTTGGATC	202	193
	A	TGAGTTGTAGGCACGGCATAG	395	
26	S	ATGTCTATACTCCGCAGATTCC	468	156
	A	ACAGTGACGACTACGATGTCCT	624	
27	S	AAAGATGGCAAACGCTACGAG	4232	174
	A	TTCAGGCATATCTCCGTTCAA	4406	
28	S	CAACGAAGGTTTTTACGGTAAA	1769	181
	A	AAAAGACTTCCTGCGGGTTC	1950	
29	S	AATGTCAAGAAAGTGGTTCGC	1290	179
	A	GTATCGCAATATCGATGGTGG	1469	
30	S	AAAAGACGAACAATAAGGACAA	1412	196
	A	AGGGAACCAGACCATAAGAGT	1608	
31	S	TAACAACCCCAAAAGTAACAGC	1597	249
	A	CGTCTAGCGAGATAGATTTAGAAG	1846	
32	S	ATTTATGCTGTGGGACAACTC	724	240
	A	GAAAGCTAGATATTAGGGAGCC	964	
33	S	CTGTGATTATGGAAGACCTGC	672	180
	A	TGGAAGAGTTTTAGTGTCGTTATA	852	
34	S	GATTCGATCTGATTTATAGCGA	314	198
	A	CAATATCTGAACCTGCACTGAC	512	
35	S	AAGACCAAGTTGCGAATAGTT	2422	145
	A	TTCCTTAGTTACAGATACCAGC	2567	
36	S	GTTCCTAACAAGAATGACCGT	354	200
	A	TTTATCCGCAACTAGGTATGC	554	
